# Supplementary material for: Lung Cancer Mortality Trends in a Brazilian City with a Long History of Asbestos Consumption
Source: Int J Environ Res Public Health. 2019 Jul 17;16(14):2548. doi: 10.3390/ijerph16142548 (PMC6679146; doi:10.3390/ijerph16142548)
Supplement: Supplementary file 1 [file ijerph-16-02548-s001.zip › Figure S1.doc]

(a) Osasco

(b) Sorocaba

(c) State of São Paulo

**Figure S1**. Age-specific rate of mortality from the trachea, bronchus and lung cancer by period and birth cohort, in female aged ≥60 years. São Paulo-Brazil 1980-2016: (**a**) Age-specific rate of mortality trachea, bronchus and lung cancer by period and birth cohort in Osasco (**b**) Age-specific rate of mortality from trachea, bronchus and lung cancer by period and birth cohort in Sorocaba (**c**) Age-specific rate of mortality from trachea, bronchus and lung cancer by period and birth cohort in State of São Paulo-Brazil.
