# Supplementary material for: Lung Cancer Mortality Trends in a Brazilian City with a Long History of Asbestos Consumption
Source: Int J Environ Res Public Health. 2019 Jul 17;16(14):2548. doi: 10.3390/ijerph16142548 (PMC6679146; doi:10.3390/ijerph16142548)
Supplement: Supplementary file 1 [file ijerph-16-02548-s001.zip › Figure S2.doc]

(a) Osasco

(b) Sorocaba

(c) State of São Paulo

**Figure S2** Age-cohort-period effects on mortality due to trachea, bronchial and lung cancer in female aged ≥60 years. São Paulo-Brazil, 1980-2016: (**a )** Age-cohort-period effects on mortality due to trachea, bronchial and lung cancer in Osasco (**b**) Age-cohort-period effects on mortality due to trachea, bronchial and lung cancer in Sorocaba (**c**) Age-cohort-period effects on mortality due to trachea, bronchial and lung cancer in São Paulo State.
