# Supplementary material for: Lung Cancer Mortality Trends in a Brazilian City with a Long History of Asbestos Consumption
Source: Int J Environ Res Public Health. 2019 Jul 17;16(14):2548. doi: 10.3390/ijerph16142548 (PMC6679146; doi:10.3390/ijerph16142548)
Supplement: Supplementary file 1 [file ijerph-16-02548-s001.zip › Figure S3.doc]

**Figure S3** Percentage of adult smokers in the city of São Paulo-Brazil, 1971-2017
