# Supplementary material for: Lung Cancer Mortality Trends in a Brazilian City with a Long History of Asbestos Consumption
Source: Int J Environ Res Public Health. 2019 Jul 17;16(14):2548. doi: 10.3390/ijerph16142548 (PMC6679146; doi:10.3390/ijerph16142548)
Supplement: Supplementary file 1 [file ijerph-16-02548-s001.zip › Table S1.doc]

**Table S1**. Age-Period-Cohort models by effect cohort for mortality due to trachea, bronchial and lung cancer , male sex aged ≥60 years . São Paulo-Brazil, 1980-2016.

| **Cohort** | **RR^1^** | **IC^2^** |
| --- | --- | --- |
| **Osasco** | | |
| 1901-1905 | 0.740 | 0.577-0.949 |
| 1906-1910 | 0.831 | 0.713-0.968 |
| 1911-1915 | 0.933 | 0.877-0.992 |
| 1916-1920 | 1.047 | 0.991-1.107 |
| 1921-1925 | 1.157 | 1.018-1.314 |
| 1926-1930 | 1.158 | 1.033-1.299 |
| 1931-1935 | 1.016 | 0.888-1.162 |
| 1936-1940 | 0.926 | 0.835-1.026 |
| 1941-1945 | 0.904 | 0.809-1.009 |
| 1946-1950 | 0.915 | 0.801-1.044 |
| 1951-1955 | 0.931 | 0.710-1.221 |
| **Sorocaba** | | |
| 1901-1905 | 0.951 | 0.745-1.214 |
| 1906-1910 | 0.953 | 0.820-1.108 |
| 1911-1915 | 0.955 | 0.899-1.015 |
| 1916-1920 | 0.957 | 0.906-1.012 |
| 1921-1925 | 0.972 | 0.857-1.102 |
| 1926-1930 | 1.062 | 0.948-1.189 |
| 1931-1935 | 1.194 | 1.042-1.369 |
| 1936-1940 | 1.020 | 0.911-1.142 |
| 1941-1945 | 0.909 | 0.811-1.020 |
| 1946-1950 | 0.881 | 0.767-1.012 |
| 1951-1955 | 0.866 | 0.645-1.161 |
| **State of São Paulo** | | |
| 1901-1905 | 0.799 | 0.740-0.862 |
| 1906-1910 | 0.932 | 0.894-0.972 |
| 1911-1915 | 0.976 | 0.947-1.006 |
| 1916-1920 | 0.997 | 0.973-1.022 |
| 1921-1925 | 1.041 | 1.020-1.063 |
| 1926-1930 | 1.095 | 1.075-1.116 |
| 1931-1935 | 1.049 | 1.031-1.067 |
| 1936-1940 | 1.020 | 1.002-1.039 |
| 1941-1945 | 0.940 | 0.921-0.960 |
| 1946-1950 | 0.897 | 0.873-0.921 |
| 1951-1955 | 0.869 | 0.834-0.906 |

^1^RR: relative risk  ^2^ IC: confidence interval of 95%.
