# Supplementary material for: Lung Cancer Mortality Trends in a Brazilian City with a Long History of Asbestos Consumption
Source: Int J Environ Res Public Health. 2019 Jul 17;16(14):2548. doi: 10.3390/ijerph16142548 (PMC6679146; doi:10.3390/ijerph16142548)
Supplement: Supplementary file 1 [file ijerph-16-02548-s001.zip › Table S2.doc]

**Table S2**. Age-Period-Cohort models by effect period for mortality due to trachea, bronchial and lung cancer, male sex aged ≥60 years. São Paulo-Brazil, 1980-2016.

| **Period** | **RR^1^** | **IC^2^** |
| --- | --- | --- |
| **Osasco** | | |
| 1981-1985 | 0.957 | 0.773-1.184 |
| 1986-1990 | 0.909 | 0.804-1.029 |
| 1991-1995 | 0.877 | 0.763-1.008 |
| 1996-2000 | 0.922 | 0.819-1.038 |
| 2001-2005 | 1.029 | 0.893-1.186 |
| 2006-2010 | 0.966 | 0.848-1.102 |
| 2011-2015 | 1.154 | 1.005-1.325 |
| **Sorocaba** | | |
| 1981-1985 | 1.183 | 0.954-1.466 |
| 1986-1990 | 1.209 | 1.068-1.368 |
| 1991-1995 | 1.202 | 1.027-1.407 |
| 1996-2000 | 1.076 | 0.954-1.212 |
| 2001-2005 | 0.975 | 0.857-1.109 |
| 2006-2010 | 0.968 | 0.847-1.107 |
| 2011-2015 | 0.857 | 0.736-0.996 |
| **State of São Paulo** | | |
| 1981-1985 | 1.009 | 0.982-1.037 |
| 1986-1990 | 1.003 | 0.979-1.028 |
| 1991-1995 | 1.067 | 1.044-1.091 |
| 1996-2000 | 1.063 | 1.043-1.084 |
| 2001-2005 | 1.049 | 1.031-1.067 |
| 2006-2010 | 0.923 | 0.908-0.938 |
| 2011-2015 | 0.968 | 0.953-0.984 |

^1^RR: relative risk  ^2^ IC: confidence interval of 95%.
