# Supplementary material for: Lung Cancer Mortality Trends in a Brazilian City with a Long History of Asbestos Consumption
Source: Int J Environ Res Public Health. 2019 Jul 17;16(14):2548. doi: 10.3390/ijerph16142548 (PMC6679146; doi:10.3390/ijerph16142548)
Supplement: Supplementary file 1 [file ijerph-16-02548-s001.zip › Table S3.doc]

**Table S3**. Age-Period-Cohort for mortality due to trachea, bronchial and lung cancer, female sex aged ≥60 years. São Paulo-Brazil, 1980-2016.

| **Model** | **Resid** | **Resid** | **Deviance^1^** | **p^2^** |
| --- | --- | --- | --- | --- |
|  | **df** | **Dev** |  |  |
| **Osasco** | | | | |
| Age | 30 | 42.993 |  |  |
| **Age-drift^3^** | **29** | **29.362** | **13.6309** | **<0.001** |
| Age-Cohort | 26 | 28.093 | 1.2694 | 0.736 |
| Age-Period-Cohort | 23 | 21.326 | 6.7674 | 0.079 |
| Age-Period | 26 | 24.064 | -2.7382 | 0.433 |
| Age-drift^4^ | 29 | 29.362 | -5.2985 | 0.151 |
| **Sorocaba** | | | | |
| Age | 30 | 25.397 |  |  |
| Age-drift^3^ | 29 | 23.976 | 1.4220 | 0.232 |
| Age-Cohort | 26 | 22.246 | 1.7295 | 0.630 |
| Age-Period-Cohort | 23 | 16.663 | 5.5828 | 0.133 |
| Age-Period | 26 | 20.101 | -3.4373 | 0.329 |
| Age-drift^4^ | 29 | 23.976 | -3.8750 | 0.275 |
| **State of São Paulo** | | | | |
| Age | 30 | 903.44 |  |  |
| Age-drift^3^ | 29 | 242.25 | 661.18 | <0.001 |
| Age-Cohort | 20 | 182.14 | 60.12 | <0.001 |
| **Age-Period-Cohort** | **15** | **33.78** | **148.35** | **<0.001** |
| Age-Period | 24 | 74.11 | -40.33 | <0.001 |
| Age-drift^4^ | 29 | 242.25 | -168.14 | <0.001 |

^1^ Deviance: Difference in degrees of freedom and deviance between the current model and the previous one, in the likelihood ratio test.

^2^ p : p-value of the likelihood ratio test.

^3^ Age-drift : Model in which the effect of age can be decomposed into period and cohort effects.

^4^ Age-drift : Model in which the longitudinal effect of age can be decomposed into effects of age and period.
